# Association of ulcerative colitis symptom severity and proctocolectomy with multidimensional patient-reported outcomes: a cross-sectional study

**DOI:** 10.1007/s00535-023-02005-7

**Published:** 2023-06-23

**Authors:** Katsuyoshi Matsuoka, Hajime Yamazaki, Masakazu Nagahori, Taku Kobayashi, Teppei Omori, Yohei Mikami, Toshimitsu Fujii, Shinichiro Shinzaki, Masayuki Saruta, Minoru Matsuura, Takayuki Yamamoto, Satoshi Motoya, Toshifumi Hibi, Mamoru Watanabe, Jovelle Fernandez, Shunichi Fukuhara, Tadakazu Hisamatsu

**Affiliations:** 1 Division of Gastroenterology and Hepatology, Department of Internal Medicine, Toho University Sakura Medical Center, 564-1 Shimoshizu, Sakura, Chiba 285-8741 Japan; 2 Section of Clinical Epidemiology, Department of Community Medicine, Graduate School of Medicine, Kyoto University, Kyoto, Japan; 3 Department of Gastroenterology and Hepatology, Tokyo Medical and Dental University, Tokyo, Japan; 4 Center for Advanced IBD Research and Treatment, Kitasato University Kitasato Institute Hospital, Tokyo, Japan; 5 Institute of Gastroenterology, Tokyo Women’s Medical University, Tokyo, Japan; 6 Division of Gastroenterology and Hepatology, Department of Internal Medicine, Keio University School of Medicine, Tokyo, Japan; 7 Department of Gastroenterology and Hepatology, Osaka University Graduate School of Medicine, Osaka, Japan; 8 Division of Gastroenterology and Hepatology, Department of Internal Medicine, The Jikei University School of Medicine, Tokyo, Japan; 9 Department of Gastroenterology and Hepatology, Kyorin University School of Medicine, Tokyo, Japan; 10 Inflammatory Bowel Disease Center and Department of Surgery, Yokkaichi Hazu Medical Center, Mie, Japan; 11 Inflammatory Bowel Disease Center, Sapporo Kosei General Hospital, Hokkaido, Japan; 12 Advanced Research Institute, Tokyo Medical and Dental University, Tokyo, Japan; 13 Japan Medical Office, Takeda Pharmaceutical Company Limited, Tokyo, Japan; 14 Department of Health Policy Management, Johns Hopkins Bloomberg School of Public Health, Maryland, USA; 15 Present Address: Division of Gastroenterology and Hepatology, Department of Internal Medicine, Hyogo Medical University, Hyogo, Japan

**Keywords:** Inflammatory bowel disease, Patient-focused registry, Work productivity

## Abstract

**Background:**

The YOu and Ulcerative colitis: Registry and Social network (YOURS) is a large-scale, multicenter, patient-focused registry investigating the effects of lifestyle, psychological factors, and clinical practice patterns on patient-reported outcomes in patients with ulcerative colitis in Japan. In this initial cross-sectional baseline analysis, we comprehensively explored impacts of symptom severity or proctocolectomy on nine patient-reported outcomes.

**Methods:**

Patients receiving tertiary care at medical institutions were consecutively enrolled in the YOURS registry. The patients completed validated questionnaires on lifestyle, psychosocial factors, and disease-related symptoms. Severity of symptoms was classified with self-graded stool frequency and rectal bleeding scores (categories: remission, active disease [mild, moderate, severe]). The effects of symptom severity or proctocolectomy on nine scales for quality of life, fatigue, anxiety/depression, work productivity, and sleep were assessed by comparing standardized mean differences of the patient-reported outcome scores.

**Results:**

Of the 1971 survey responses analyzed, 1346 (68.3%) patients were in remission, 583 (29.6%) had active disease, and 42 (2.1%) had undergone proctocolectomy. A linear relationship between increasing symptom severity and worsening quality of life, fatigue, anxiety, depression, and work productivity was observed. Patients with even mild symptoms had worse scores than patients in remission. Patients who had undergone proctocolectomy also had worse scores than patients in remission.

**Conclusions:**

Ulcerative colitis was associated with reduced mood, quality of life, fatigue, and work productivity even in patients with mild symptoms, suggesting that management of active ulcerative colitis may improve patient-reported outcomes irrespective of disease severity. (UMIN Clinical Trials Registry: UMIN000031995, https://www.umin.ac.jp/ctr/index-j.htm).

**Supplementary Information:**

The online version contains supplementary material available at 10.1007/s00535-023-02005-7.

## Introduction

Ulcerative colitis (UC) is a chronic relapsing and remitting inflammatory bowel disease (IBD) of the colon and rectum [1–3]. The majority of patients first present with symptoms of UC between their late 10 s and early 30 s [1]. The hallmark feature of active UC is bloody diarrhea, often accompanied by abdominal pain [1, 2]. To avoid unwanted clinical outcomes, including relapse, hospitalization, and colectomy, patients need to continue treatment throughout their lives with good communication and support from their healthcare providers (HCPs).

A major therapeutic target in IBD involves addressing and improving the patient’s overall burden of disease [4]. UC symptoms are distressing for patients, and the disease affects relationships, careers, and family life, with significant psychosocial impacts reported even in patients with ‘controlled’ symptoms [5, 6]. In a French study involving the administration of six validated questionnaires on patient-reported outcomes (PROs) for patients with IBD, approximately half reported disease-induced depression and low quality of life (QOL) [7]. In another study, one-third of UC patients reported that they felt anxious and stigmatized [8]. Using the United States and European Union 5 data from the 2015 and 2017 Adelphi Inflammatory Bowel Disease-Specific Programme, a recent retrospective study in patients with moderate-to-severe UC demonstrated that active UC was significantly associated with reduced health-related QOL and leisure- and work-related impairment [9]. Thus, evaluating QOL, work productivity, fatigue, disability, anxiety, and depression by PROs is now recognized as an essential element in the management of IBD, and it is important for both patients and their HCPs to understand how the burden of disease may change with disease presentation [2, 7, 9].

Four key categories of modifiable factors to consider in improving patient outcomes have been identified: lifestyle (diet, physical activity, sleep, work); psychosocial factors (stress, depression, social support); practice patterns; and gaps between patients’ needs and HCPs’ practice [10–15]. The degree to which each of these factors influences outcomes in patients with UC is uncertain and the optimal lifestyle, psychosocial support, and practice patterns for this group of patients have been debated. The YOu and Ulcerative colitis: Registry and Social network (YOURS) is a large-scale, observational study investigating the effect of lifestyle, psychological factors, and clinical practice patterns on PROs and hospitalization and colectomy rates over 3 years in patients with UC in Japan [16]. The YOURS is a patient-focused registry that allows patients to access and compare their own data with summarized data from other patients. Patient-focused registries may improve healthcare by enabling patients, HCPs, and scientists to co-produce better health outcomes and support practice-based improvement [17]. Here, we report data focused on the impact of disease-related symptoms and proctocolectomy on PROs from the initial baseline survey for patients enrolled in the YOURS registry.

## Methods

### Study design

In this cross-sectional analysis, baseline data from the YOURS registry, a multicenter prospective, observational study, were analyzed [16]. A detailed description of the registry has been previously reported [16]. Briefly, the YOURS registry enrolled 2006 patients who have been diagnosed with UC according to the Evidence-Based Clinical Practice Guidelines for Inflammatory Bowel Disease [1]. Patients were enrolled during visits at five core IBD hospitals in Japan from May 2018 to January 2019. Enrolled patients were asked to complete surveys at the initial visit, 3 months after the initial visit (if in remission), and each year for up to 3 years after the initial visit. A three-item brief symptom survey was also conducted every 3 months after the initial visit. The protocol of this study has been posted to the UMIN Clinical Trials Registry (UMIN000031995).

Lifestyle, psychosocial factors, clinical practice patterns, and gaps between patient need and HCP practice were all assessed for their effects on PROs and unfavorable clinical outcomes, including relapse/exacerbation, hospitalization, and colectomy. Data collected at the initial visit were analyzed in this study.

Upon request, patients who participated in YOURS were given access to the registry website (https://ibd.pedal.or.jp/) where they could review their data over time, compare their data with other patients, and share their data with healthcare professionals, provided the patients have given written consent to transferring their data to the website.

### Patient selection

Eligible patients had a diagnosis of UC, were aged ≥ 16 years at informed consent and were attending one of the five investigational sites. Consecutive enrolment commenced at each investigational site following approval by their respective ethics committee and continued until 2000 patients were enrolled in total across the sites or on 31 December 2018, whichever came later.

### Survey items

**Fig. 1:**
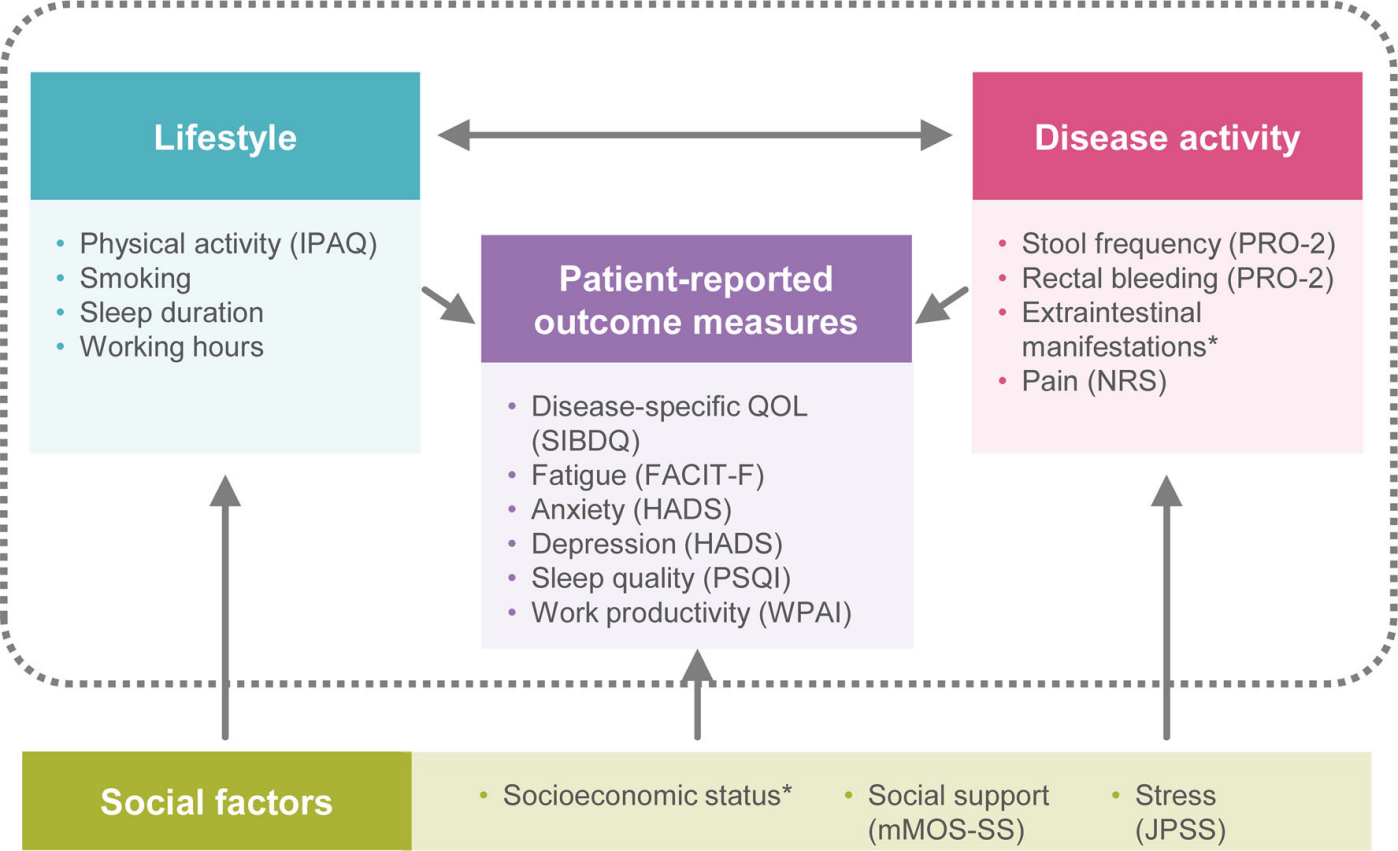
YOURS Registry: Study Concept. *Collected from patient charts. *FACIT-F* Functional Assessment of Chronic Illness Therapy – Fatigue, *HADS* Hospital Anxiety and Depression Scale, *IPAQ* International Physical Activity Questionnaire, *JPSS* Japanese version of the Perceived Stress Scale, *mMOS-SS* modified Medical Outcomes Study Social Support Survey, *NRS* Numerical Rating Scale, *PRO-2* Two-item Patient Reported Outcomes, *PSQI* Pittsburgh Sleep Quality Index, *QOL* quality of life, *SIBDQ* Short Inflammatory Bowel Disease Questionnaire, *WPAI* Work Productivity and Activity Impairment

At their initial visit, patients completed written questionnaire surveys with questions on demographic information, disease activity, disease characteristics (disease history, extraintestinal manifestations, abdominal pain, current and previous medication), socioeconomic status (employment, annual income, education, and social factors), lifestyle factors (physical activity, smoking history, sleep, and work), and PROs (Fig. 1).

Remission was defined on the basis of the Two-Item Patient Reported Outcomes (PRO-2) questionnaire [18] as having a stool frequency score of 0 or 1 and a rectal bleeding score of 0 (total PRO-2 score of 0 or 1) [18, 19]. All patients not in remission were deemed as having active disease, and symptom severity was defined according to total PRO-2 score: 1 (excluding those in remission) or 2, mild; 3 or 4, moderate; and 5 or 6, severe.

As a disease characteristic, abdominal pain was measured by Numerical Rating Scale (NRS) score [20], which ranges from 0 (no pain at all) to 10 (worst pain ever possible). Social factors were assessed by the modified Medical Outcomes Study Social Support Survey (mMOS-SS) [21, 22] and the Japanese version of the Perceived Stress Scale (JPSS) [23, 24]. Higher transformed mMOS-SS scores (range 0–100) reflected stronger social support. The sum of JPSS sub-scores (range 0–56) reflected more stress with higher scores. As a lifestyle factor, physical activity was measured by the International Physical Activity Questionnaire (IPAQ) [25, 26] and higher total Metabolic Equivalent Task (MET) score corresponds to higher physical activity (range 0–19,278). Physical activity was categorized as high, moderate, or low based on frequency, intensity, and length of activity and total MET [27]. Brinkman index was defined by the number of cigarettes smoked per day multiplied by the number of years of smoking [28].

PROs were assessed by using the following instruments: Short Inflammatory Bowel Disease Questionnaire (SIBDQ) [29, 30], Functional Assessment of Chronic Illness Therapy – Fatigue (FACIT-F) [31], Hospital Anxiety and Depression Scale (HADS) [32–34], Work Productivity and Activity Impairment (WPAI) [35], and Pittsburgh Sleep Quality Index (PSQI) [36, 37]. Ranges of SIBDQ sub-scores (divided by 10) for bowel symptoms, systemic symptoms, emotional function, and social function were 0.3–2.1, 0.2–1.4, 0.3–2.1, 0.2–1.4 (total score 1–7), respectively, with higher scores reflecting better IBD-related QOL. The FACIT-F score (range 0–52) of < 30 indicates severe fatigue [31, 38]. For the HADS scores for anxiety and depression (range 0–21 for each), the severity classes were defined as: normal, 0–7; mild, 8–10; moderate, 11–14; and severe, 15–21 [33]. WPAI outcomes were expressed as % of impairment, with higher numbers indicating greater impairment and less work productivity; the degree of impairment was defined as: mild 0–19%; moderate 20–49%; and severe ≥ 50% [35]. The PSQI contains 19 self-rated questions combined to form 7 component scores, each with a range of 0–3 (0, no difficulty; 3, severe difficulty) to yield a total PSQI score (range 0–21), with a higher score indicating lower sleep quality and a score > 5 indicating poor sleep quality [36].

### Statistical analysis

Analysis groups were determined for each survey item and outcome measures. Descriptive statistics for demographic, lifestyle, and PRO data were computed for remission, active disease (mild, moderate, severe, and total), and post-proctocolectomy. Quantitative variables were summarized by the median (interquartile range [IQR]), and qualitative variables were expressed as the number (%). To compare the effect of symptom severity and proctocolectomy on the PROs, the standardized mean difference (SMD) [39] of the PROs and their 95% confidence intervals were computed using remission as the comparator, with the following formula:

$$\text{SMD} =\frac{\text{PRO score} -\text{PRO score of remission}}{\text{standard deviation of remission}}.$$

The magnitude of the effect size was interpreted as: small, SMD = 0.2; medium, SMD = 0.5; and large, SMD = 0.8 [39]. In patients in remission and those with active disease, the variance of each PRO was modelled by linear regression and the fraction of variance explained by symptom severity was calculated. Correlations among pairs of PROs were assessed by calculating Spearman correlation coefficients in patients in remission and those with active disease who were assessable. For two-dimensional hierarchical clustering analysis, patients in remission and those with active disease who were assessable for all nine PROs were included. The PRO scores were standardized by subtracting the variable’s mean from each observed score, then dividing it by the variable’s standard deviation. For SIBDQ and FACIT-F, the sign of the standardized scores was flipped so that negative and positive values would correspond to better and worse symptoms, respectively. Euclidian distance between the variables was computed and clustered using the complete linkage algorithm.

The total number of analyzed patients and the number of missing cases were reported for each variable in the analysis. Imputation of missing data and data cleaning were carried out according to the instructions for each validated questionnaire. All analyses were conducted using SAS version 9.4 32-bit. Heatmaps and hierarchical clustering dendrograms were generated using heatmap.2 function from the gplots package (version 3.1.0) in R version 3.6.3.

### Ethics approval

This study was conducted in accordance with the 1964 Declaration of Helsinki and its later amendments and all applicable Japanese laws and guidelines. The study was approved by the ethics committees of the following five investigational sites (approval number) prior to study start: Tokyo Medical and Dental University, Medical Hospital (M2017-327-10); Kitasato University Kitasato Institute Hospital (18010); Kyorin University Hospital (1096); Tokyo Women's Medical University Hospital (4817); and Toho University Sakura Medical Center (S18043). All participants gave written informed consent before participating in this study. The study was registered with the UMIN Clinical Trials Registry (UMIN000031995) before enrolment of the first patient.

## Results

### Patient demographics by disease activity and history of proctocolectomy

Of a total of 2731 UC patients attending the participating centers, 2006 (73.5%) patients were enrolled in the study. Of 1971 patients whose data were analyzed (Supplementary Fig. 1), 1346 (68.3%) patients were in remission, 583 (29.6%) had active disease, and 42 (2.1%) had undergone proctocolectomy. Patient demographics and disease characteristics are shown in Table 1. The median age of patients who were in remission or had active disease was 44.0 years and 42.0 years, respectively, and 53–56% of these patients were male. Patients who had undergone proctocolectomy had a median age of 45.0 years, were 78.6% male, and 12 patients received ongoing treatment with immunomodulators (*n* = 2), tumor necrosis factor (TNF)-alpha inhibitors (*n* = 6), systemic steroids (*n* = 5), tofacitinib (*n* = 1), and vedolizumab (*n* = 1).

**Table 1:** Patient demographics and disease characteristics

Characteristics	Remission (*n* = 1346)	Active disease (*n* = 583)	Post proctocolectomy (*n* = 42)
Demographics			
Age (years), median (IQR)^a^	44 (21)	42 (21)	45 (18)
Sex, *n* (%)			
Male	711 (52.8)	327 (56.1)	33 (78.6)
Female	633 (47.0)	255 (43.7)	9 (21.4)
Missing	2 (0.1)	1 (0.2)	0
BMI, median (IQR)^b^	21.7 (4.2)	21.6 (4.7)	20.9 (4.3)
Disease characteristics			
Age at diagnosis (years), median (IQR)^c^	33 (21)	30 (19)	34 (24)
Disease duration (years), median (IQR)^d^	9.3 (11.0)	9.0 (12.2)	11.3 (13.4)
Disease extent, *n* (%)			
Extensive colitis	735 (54.6)	291 (49.9)	35 (83.3)
Left-sided colitis	324 (24.1)	172 (29.5)	6 (14.3)
Proctitis	245 (18.2)	101 (17.3)	0
Right-sided colitis	20 (1.5)	4 (0.7)	0
Unknown	18 (1.3)	6 (1.0)	1 (2.4)
Missing	4 (0.3)	9 (1.5)	0
Extraintestinal manifestations, *n* (%)			
Peripheral arthropathy	41 (3.0)	18 (3.1)	3 (7.1)
Erythema nodosum	8 (0.6)	4 (0.7)	0
Primary sclerosing cholangitis	7 (0.5)	2 (0.3)	0
Aphthous stomatitis	6 (0.4)	3 (0.5)	0
Pancreatitis	6 (0.4)	3 (0.5)	0
Pyoderma gangrenosum	6 (0.4)	2 (0.3)	0
Spine arthropathy	5 (0.4)	3 (0.5)	1 (2.4)
Iritis	1 (0.1)	1 (0.2)	0
Ankylosing spondylitis	1 (0.1)	0	0
Scleritis	1 (0.1)	0	0
Other skin lesion	31 (2.3)	15 (2.6)	0
Missing	0	1 (0.2)	0
Abdominal pain (NRS), median (IQR)^e^	0 (1)	1 (3)	0 (3)
Current medication, *n* (%)			
5-ASA (oral)	1154 (85.7)	496 (85.1)	12 (28.6)
Immunomodulators	331 (24.6)	135 (23.2)	2 (4.8)
TNF-alpha inhibitors	219 (16.3)	124 (21.3)	6 (14.3)
Systemic steroids (prednisolone)	59 (4.4)	39 (6.7)	5 (11.9)
Calcineurin inhibitors	16 (1.2)	19 (3.3)	0
Tofacitinib	15 (1.1)	18 (3.1)	1 (2.4)
Apheresis	5 (0.4)	1 (0.2)	0
Vedolizumab	2 (0.1)	3 (0.5)	1 (2.4)

*5-ASA* 5-aminosalicylic acid, *BMI* body mass index, *IQR* interquartile range, *NRS* numerical rating scale, *TNF* tumor necrosis factor

^a^Remission (*n* = 1344), Active disease (*n* = 583), Post proctocolectomy (*n* = 42)

^b^Remission (*n* = 1340), Active disease (*n* = 583), Post proctocolectomy (*n* = 42)

^c^Remission (*n* = 1288), Active disease (*n* = 564), Post proctocolectomy (*n* = 41)

^d^Remission (*n* = 1290), Active disease (*n* = 566), Post proctocolectomy (*n* = 41)

^e^Remission (*n* = 1343), Active disease (*n* = 583), Post proctocolectomy (*n* = 42)

### Socioeconomic, educational, and social factors by disease activity and history of proctocolectomy

Socioeconomic and social factors are shown in Table 2 and Supplementary Table 1. Approximately 70% of patients in remission, those who had active disease, and those who had undergone proctocolectomy were in paid employment. Among patients with active disease, 22.4%, 20.0%, and 38.5% of those with mild, moderate, or severe symptoms, respectively, were unemployed. The proportion of patients with an annual income of JPY < 5 million (approximately USD 46,300) was 36.2% of patients in remission; 44.9% of patients with active disease, including 47.2%, 37.9%, and 65.4% of patients with mild, moderate, and severe symptoms, respectively; and 52.3% of patients who had undergone proctocolectomy.

**Table 2:** Social and lifestyle factors by disease activity

Factors	Remission (*n* = 1346)	Active disease (*n* = 583)	Post proctocolectomy (*n* = 42)
Socioeconomic status			
Employment, *n* (%)			
Student	91 (6.8)	44 (7.5)	2 (4.8)
Unemployed	275 (20.4)	130 (22.3)	12 (28.6)
Employed	971 (72.1)	407 (69.8)	28 (66.7)
Missing	9 (0.7)	2 (0.3)	0
Annual income (JPY), *n* (%)			
< 3 million	160 (11.9)	95 (16.3)	8 (19.0)
≥ 3 and < 5 million	327 (24.3)	167 (28.6)	14 (33.3)
≥ 5 and < 7 million	278 (20.7)	111 (19.0)	6 (14.3)
≥ 7 and < 10 million	260 (19.3)	102 (17.5)	8 (19.0)
≥ 10 and < 12 million	131 (9.7)	36 (6.2)	4 (9.5)
≥ 12 million	145 (10.8)	62 (10.6)	2 (4.8)
Missing	45 (3.3)	10 (1.7)	0
Education, *n* (%)			
Junior high school	22 (1.6)	16 (2.7)	0
High school	217 (16.1)	107 (18.4)	11 (26.2)
Vocational school	171 (12.7)	85 (14.6)	6 (14.3)
Community college	117 (8.7)	39 (6.7)	2 (4.8)
University	535 (39.7)	219 (37.6)	18 (42.9)
Graduate school	82 (6.1)	31 (5.3)	2 (4.8)
Missing	202 (15.0)	86 (14.8)	3 (7.1)
Social factors			
Social support (mMOS-SS), median (IQR)^a^	75.00 (31.25)	71.88 (37.50)	68.75 (43.75)
Stress (JPSS), median (IQR)^b^	23 (9)	26 (11)	25 (10)
Living alone, *n* (%)	211 (15.7)	95 (16.3)	12 (28.6)
Physical activity (IPAQ-short)			
Total MET (minutes/week), median (IQR)^c^	1386 (2338)	1188 (2346)	1164 (1683)
Total MET (minutes/week), *n* (%)			
Low	122 (9.1)	55 (9.4)	7 (16.7)
Moderate	976 (72.5)	428 (73.4)	29 (69.0)
High	217 (16.1)	85 (14.6)	5 (11.9)
Missing	31 (2.3)	15 (2.6)	1 (2.4)
Smoking history			
Brinkman index, median (IQR)^d^	205 (300)	200 (350)	300 (260)
Smoking status, *n* (%)			
Never	825 (61.3)	358 (61.4)	19 (45.2)
Current	115 (8.5)	46 (7.9)	3 (7.1)
Former	405 (30.1)	179 (30.7)	20 (47.6)
Missing	1 (0.1)	0	0
Sleep, median (IQR)			
Sleep duration (h)^e^	6.5 (1.0)	6.0 (1.0)	6.5 (1.0)
Work, median (IQR)			
Work hour (h/week)^f^	40 (22)	40 (20)	40 (27)

*IPAQ* International Physical Activity Questionnaire, *IQR* interquartile range, *JPSS* Japanese version of the Perceived Stress Scale, *MET* metabolic equivalent task, *mMOS-SS* modified Medical Outcomes Study Social Support Survey

^a^Remission (*n* = 1342), Active disease (*n* = 581), Post proctocolectomy (*n* = 41)

^b^Remission (*n* = 1328), Active disease (*n* = 578), Post proctocolectomy (*n* = 41)

^c^Remission (*n* = 1315), Active disease (*n* = 568), Post proctocolectomy (*n* = 41)

^d^Remission (*n* = 512), Active disease (*n* = 218), Post proctocolectomy (*n* = 23)

^e^Remission (*n* = 1343), Active disease (*n* = 583), Post proctocolectomy (*n* = 42)

^f^Remission (*n* = 964), Active disease (*n* = 405), Post proctocolectomy (*n* = 28)

Education profiles were similar across patient groups (remission, active disease, post-proctocolectomy); however, the proportion of patients whose highest level of educational attainment was high school or junior high school was disproportionally high in patients with severe symptoms.

Stress in patients with active disease increased with increasing symptom severity, with median values on the JPSS of 25, 26, and 29 for patients with mild, moderate, and severe symptoms, respectively. Levels of social support were lowest for patients with severe symptoms and for those who had undergone proctocolectomy, with median mMOS-SS values of 68.75, compared with values of 75.00 for patients in remission and 71.88 for patients with mild or moderate symptoms (Supplementary Table 1). The proportion of patients living alone was 15.7% of patients in remission, 16.3% of patients with active disease (mild, moderate, or severe), and 28.6% of patients who had undergone proctocolectomy. Among patients with active disease, 30.8% of patients with severe symptoms lived alone compared with 16.0% and 14.9% of patients with mild or moderate symptoms, respectively.

### Lifestyle factors

Levels of physical activity, sleep and work in the remission, active disease, and post-proctocolectomy patient groups are shown in Table 2. On the basis of the IPAQ, the median total MET (minutes/week) of physical exercise was 1386 in the remission group, 1188 in patients with active disease, and 1164 in the post-proctocolectomy group. In terms of exposure to smoking, 61.3% of patients in remission, 61.4% of patients with active disease, and 45.2% of patients who had undergone proctocolectomy had never smoked. Patients in the remission group slept for a median of 6.5 h per day, as did those in the post-proctocolectomy group. Patients who had active disease slept for a median of 6.0 h per day. The median number of work hours per week was 40.0 h for all patient groups.

### Patient-reported outcomes

**Fig. 2:**
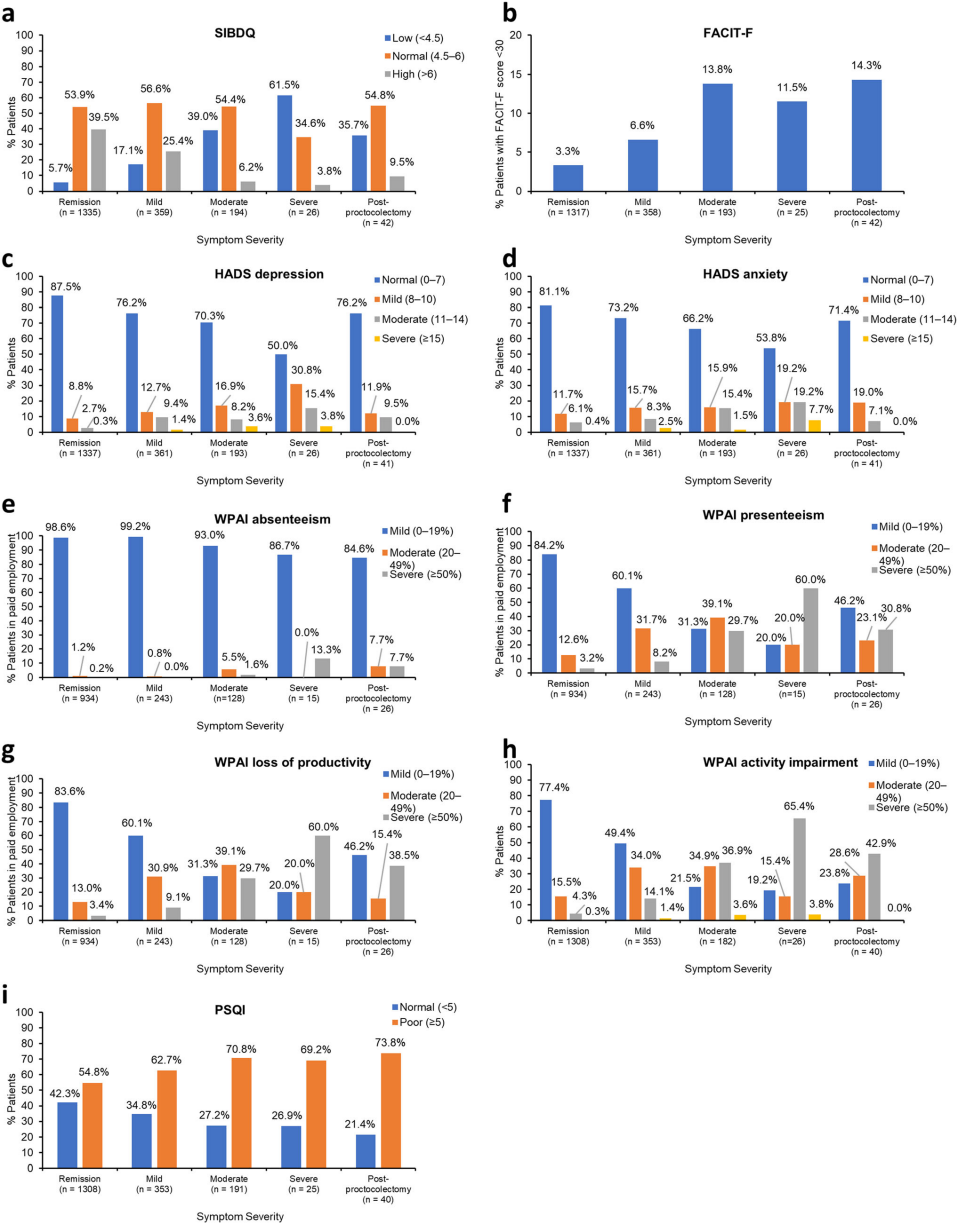
Proportion of patients reporting **a** QOL as low, normal or high on SIBDQ; **b** severe fatigue on FACIT-F; **c** depression or **d** anxiety on the HADS scale; **e** absenteeism, **f** presenteeism, **g** work productivity, and **h** activity impairment on WPAI; and **i** poor sleep quality on PSQI. *FACIT-F* Functional Assessment of Chronic Illness Therapy – Fatigue, *HADS* Hospital Anxiety and Depression Scale, *PSQI* Pittsburgh Sleep Quality Index, *QOL* quality of life, *SIBDQ* Short Inflammatory Bowel Disease Questionnaire, *WPAI* Work Productivity and Activity Impairment

Median total SIBDQ scores were 5.9, 5.2, and 5.0 in patients in remission, patients with active disease, and those who had undergone proctocolectomy, respectively (Table 3). QOL was assessed as low in 17.1% of patients with a mild symptom score, 39.0% of those with a moderate symptom score, and 61.5% of those with a severe symptom score, versus 5.7% and 35.7% of patients in remission and those who had undergone proctocolectomy, respectively (Fig. 2a).

**Table 3:** Patient-reported outcomes by symptom severity

Median (IQR)	Remission (*n* = 1346)	Active disease				Post proctocolectomy (*n* = 42)
		Mild (*n* = 362)	Moderate (*n* = 195)	Severe (*n* = 26)	Total (*n* = 583)	
SIBDQ						
Bowel symptoms^a^	1.8 (0.3)	1.7 (0.4)	1.4 (0.5)	1.2 (0.8)	1.6 (0.5)	1.7 (0.5)
Systemic symptoms^b^	1.1 (0.3)	1.0 (0.4)	0.9 (0.4)	0.8 (0.5)	1.0 (0.4)	1.0 (0.3)
Emotional function^c^	1.7 (0.4)	1.5 (0.5)	1.3 (0.5)	1.1 (0.5)	1.5 (0.5)	1.35 (0.7)
Social function^d^	1.4 (0.1)	1.3 (0.3)	1.1 (0.4)	0.9 (0.4)	1.2 (0.4)	1.0 (0.6)
Total (range 1–7)^e^	5.9 (1.0)	5.4 (1.3)	4.7 (1.5)	4.15 (1.8)	5.2 (1.4)	5.0 (1.4)
FACIT-F (range 0–52)^f^	44 (9)	42 (11)	39 (11)	36 (9)	40 (11.5)	38 (9)
HADS (range 0–21)^g^						
Depression	3 (4)	4 (5)	5 (6)	7.5 (5)	4 (6)	6 (3)
Anxiety	4 (5)	5 (6)	5 (5)	7 (7)	5 (5)	6 (5)
WPAI in %^h^						
Absenteeism	0 (0)	0 (0)	0 (0)	0 (8.7)	0 (0)	0 (0)
Presenteeism	0 (10)	10 (20)	30 (40)	60 (60)	20 (30)	20 (40)
Work productivity loss	0 (10)	10 (20)	30 (41.5)	60 (61.3)	20 (31.5)	20 (52.4)
Activity impairment^i^	0 (10)	10 (30)	30 (40)	60 (50)	20 (30)	40 (35)
PSQI total (range 0–21)^j^	5 (4)	6 (4)	6 (5)	7 (5)	6 (4)	7 (4)

*FACIT-F* Functional Assessment of Chronic Illness Therapy – Fatigue, *HADS* Hospital Anxiety and Depression Scale, *IQR* interquartile range, *PSQI* Pittsburgh Sleep Quality Index, *SIBDQ* Short Inflammatory Bowel Disease Questionnaire, *WPAI* Work Productivity and Activity Impairment

^a^Remission (*n* = 1338), Mild (*n* = 362), Moderate (*n* = 195), Severe (*n* = 26), Total (*n* = 583), Post proctocolectomy (*n* = 42)

^b^Remission (*n* = 1340), Mild (*n* = 362), Moderate (*n* = 194), Severe (*n* = 26), Total (*n* = 582), Post proctocolectomy (*n* = 42)

^c^Remission (*n* = 1341), Mild (*n* = 362), Moderate (*n* = 195), Severe (*n* = 26), Total (*n* = 583), Post proctocolectomy (*n* = 42)

^d^Remission (*n* = 1342), Mild (*n* = 359), Moderate (*n* = 195), Severe (*n* = 26), Total (*n* = 580), Post proctocolectomy (*n* = 42)

^e^Remission (*n* = 1335), Mild (*n* = 359), Moderate (*n* = 194), Severe (*n* = 26), Total (*n* = 579), Post proctocolectomy (*n* = 42)

^f^Remission (*n* = 1317), Mild (*n* = 358), Moderate (*n* = 193), Severe (*n* = 25), Total (*n* = 576), Post proctocolectomy (*n* = 42)

^g^Remission (*n* = 1337), Mild (*n* = 361), Moderate (*n* = 193), Severe (*n* = 26), Total (*n* = 580), Post proctocolectomy (*n* = 41)

^h^Remission (*n* = 934), Mild (*n* = 243), Moderate (*n* = 128), Severe (*n* = 15), Total (*n* = 386), Post proctocolectomy (*n* = 26)

^i^Remission (*n* = 1308), Mild (*n* = 353), Moderate (*n* = 182), Severe (*n* = 26), Total (*n* = 561), Post proctocolectomy (*n* = 40)

^j^Remission (*n* = 1308), Mild (*n* = 353), Moderate (*n* = 191), Severe (*n* = 25), Total (*n* = 569), Post proctocolectomy (*n* = 40)

Patients in remission had a median FACIT-F score of 44.0. In patients with active disease and those who had undergone proctocolectomy, median FACIT-F scores were 40.0 and 38.0, respectively (Table 3). The level of fatigue was assessed as severe in 6.6%, 13.8%, and 11.5% of patients with mild, moderate, and severe symptom scores, and 3.3% and 14.3% of patients in remission and those who had undergone proctocolectomy, respectively (Fig. 2b).

The median depression and anxiety sub-scores of HADS were 3.0 and 4.0, respectively, for patients in remission, versus 4.0 and 5.0, respectively, for patients with active disease, and 6.0 for both scores in patients who had undergone proctocolectomy (Table 3). HADS scores indicated that approximately 22% of patients with active disease and a mild symptom score had mild to moderate depression, and 24% had mild to moderate anxiety (Fig. 2c and d).

Responses to the WPAI questionnaire for patients in paid employment indicated severe absenteeism of 50% or more in 0.2% of patients in remission, 0%, 1.6%, and 13.3% of patients with mild, moderate, and severe symptom scores, respectively, and 7.7% of patients who had undergone proctocolectomy (Fig. 2e). Severe presenteeism (≥ 50%) was reported by 8.2%, 29.7%, and 60.0% of patients with mild, moderate, and severe symptom scores, and 3.2% and 30.8% of patients in remission and those who had undergone proctocolectomy (Fig. 2f). Severe work productivity loss (≥ 50%) was reported by 3.4% of patients in remission, 9.1%, 29.7%, and 60.0% of patients with mild, moderate, and severe symptom scores, respectively, and 38.5% of patients who had undergone proctocolectomy (Fig. 2g). In all patients irrespective of employment status, severe activity impairment of 50% or more was reported by 4.3% of patients in remission, 14.1%, 36.9%, and 65.4% of patients with mild, moderate, and severe symptom scores, respectively, and 42.9% of patients who had undergone proctocolectomy (Fig. 2h). According to the PSQI, poor sleep quality was experienced by 54.8% of patients in remission, 62.7–70.8% of patients with active disease, and 73.8% of patients who had undergone proctocolectomy (Fig. 2i).

### Multidimensional PROs and UC disease activity

**Fig. 3:**
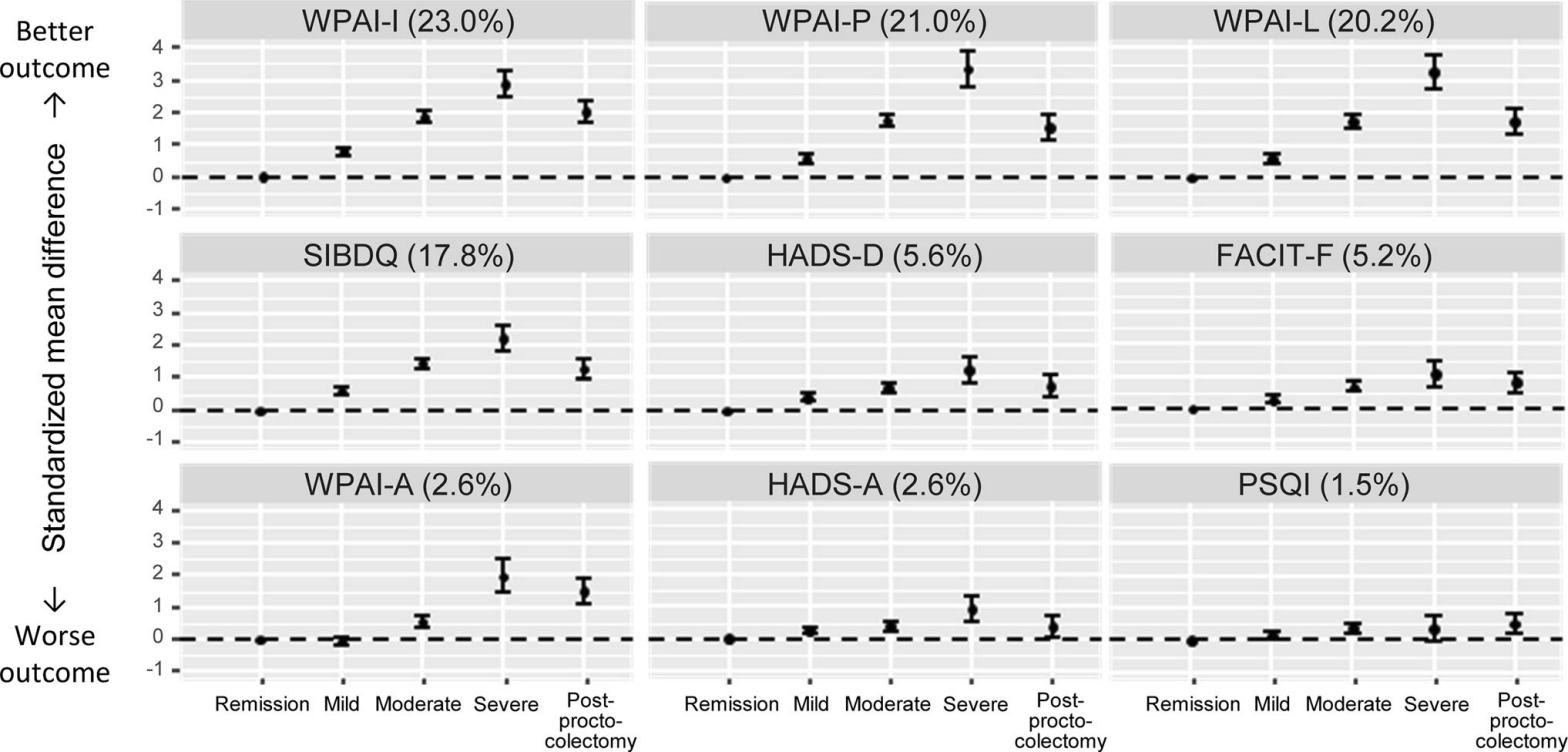
Associations of symptom severity and proctocolectomy with patient-reported outcomes. Data indicate SMD and their 95% confidence intervals. The fraction of variance of symptom severity explained by each outcome is shown in parentheses. The magnitude of the effect size was interpreted as: small, SMD = 0.2; medium, SMD = 0.5; and large, SMD = 0.8 [39]. Total number of patients included in the analysis were: SIBDQ (*N* = 1956), FACIT-F (*N* = 1935), HADS-D (*N* = 1958), HADS-A (*N* = 1958), WPAI-A (*N* = 1346), WPAI-P (*N* = 1346), WPAI-L (*N* = 1346), WPAI-I (*N* = 1909), and PSQI (*N* = 1917) (see Table 3 footnotes for breakdown by patient group). *FACIT-F* Functional Assessment of Chronic Illness Therapy – Fatigue, *HADS* Hospital Anxiety and Depression Scale (A, anxiety; D, depression), *PSQI* Pittsburgh Sleep Quality Index, *SIBDQ* Short Inflammatory Bowel Disease Questionnaire, *SMD* standardized mean difference, *WPAI* Work Productivity and Activity Impairment (A, absenteeism; I, impairment of activity; L, loss of productivity; P, presenteeism)

Linear correlations were revealed between symptom severity and most PRO scores (Fig. 3). The magnitude of the impact of symptom severity varied widely among the nine PROs. Three WPAI sub-scores had the largest variance explained by symptom severity: activity impairment (23.0%), presenteeism (21.0%), and work productivity loss (20.2%) (Fig. 3). These three WPAI sub-scores showed strongest Spearman correlations (0.85–0.99) among one another (Table 4). Other PROs also showed strong positive correlations (FACIT-F and SIBDQ; and depression and anxiety) or negative correlations (WPAI impairment and SIBDQ; and depression and fatigue) (Table 4).

**Table 4:**
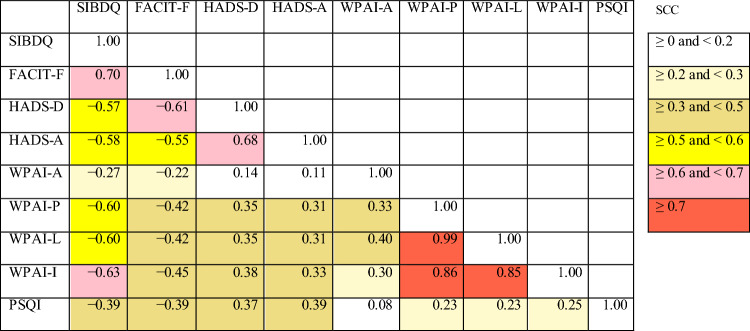
Spearman coefficients of correlation between patient-reported outcomes

The analysis included patients in remission and those with active disease (mild, moderate, or severe) who were assessable for all nine PROs (*N* = 1288)

FACIT-*F* Functional Assessment of Chronic Illness Therapy – Fatigue, *HADS* Hospital Anxiety and Depression Scale (A, anxiety; D, depression), *PRO* patient-reported outcome, *PSQI* Pittsburgh Sleep Quality Index, *SIBDQ* Short Inflammatory Bowel Disease Questionnaire, *SCC* Spearman coefficients of correlation, *WPAI* Work Productivity and Activity Impairment (A, absenteeism; I, impairment of activity; L, loss of productivity; P, presenteeism)

In all nine measures, patients who had undergone proctocolectomy had scores closer to those obtained in patients in remission, but the scores did not completely revert (Fig. 3). Even a mild symptom score yielded a medium to large effect size (SMD ≥ 0.5) on SIBDQ and WPAI (activity impairment, presenteeism, and work productivity loss), whereas the effect size of PSQI and FACIT-F was small (SMD < 0.5) in patients with mild symptoms. Severe symptoms yielded an SMD of > 1.9 on WPAI subscales and 2.2 on SIBDQ (Fig. 3).

### Hierarchical clustering analysis of PROs

To characterize the profiles of the PROs in patients in remission and those with active disease (mild, moderate, and severe), the nine PROs were clustered based on the Euclidian distances of their standardized scores using hierarchical clustering analysis. The four WPAI sub-scores clustered into one group (left side of the heatmap, Supplementary Fig. 2) and PSQI, HADS, FACIT-F, and SIBDQ into another (right side of the heatmap, Supplementary Fig. 2). Most patients with moderate or severe symptoms had worse PRO scores as did some patients with mild or no symptoms.

## Discussion

The YOURS study is the first large-scale, prospective study assessing lifestyle and PROs in approximately 2000 patients with UC in Japan. In this initial baseline analysis, we found that UC is associated with reduced mood, QOL, fatigue, and work productivity even in patients with mild symptoms. The magnitude of the impact of UC on PROs increased with symptom severity and varied depending on the PRO assessed. PRO scores were favorable in patients in remission versus patients who had undergone proctocolectomy. UC symptom severity had the greatest magnitude of impact on WPAI among the PROs on a standardized scale, suggesting that decreased work productivity may represent a major challenge for working UC patients. Our results highlight the importance of monitoring work productivity in patients with UC to inform on treatment strategies to reduce the disease burden in patients.

Expectedly, QOL, as measured by the SIBDQ, was lower in patients with active disease than in those in remission. For patients in the active disease state, a worsening trend in QOL was observed for all measured SIBDQ domains, including bowel and systemic symptoms and emotional and social functioning, with increasing symptom severity. These findings echo the findings of recent research in the USA and Europe [9]. Our results showed that median SIBDQ scores were favorable in patients who had undergone proctocolectomy versus those with moderate-to-severe active disease, consistent with a recent meta-analysis showing that ileal pouch–anal anastomosis is associated with improved QOL [40]. This suggests that surgery remains an important intervention option for patients with moderate-to-severe symptoms and highlights the importance of surgical management in improving physical symptoms as well as QOL.

In line with previous studies in Austria, the USA, and Europe [9, 41], our data indicate that work productivity is substantially affected by UC, as reflected by the WPAI findings. In our study, the mean absenteeism increased as symptom severity increased (data not shown). Importantly, work productivity significantly decreased in patients with increasing symptom severity even though the work hours per week were similar across the patient groups. The loss of work productivity is inevitably associated with financial and societal costs. An important finding of our study is that even patients with mild symptom severity reported reductions in work productivity. Patients with severe symptoms, who reported the greatest loss in work productivity, were over-represented in the lower income or unemployed groups. Moreover, as symptom severity increased, social support decreased and stress and living alone increased. These results imply significant burden of the disease on the patient’s financial status and social life. Therefore, physicians must recognize the effect of UC symptoms on work productivity and its impact on their patients.

Our study also found that symptom severity correlates with mood. We found a linear relationship between increasing symptom severity and moderate decreases in HADS or FACIT-F scores. In a recent nationwide prospective cohort study conducted in Korea, significant mood disorders requiring psychological interventions (HADS score ≥ 11) were identified in 17–21% of patients with UC, but there was no significant difference in the mean HADS score according to symptom severity [42]. Villoria and colleagues found that fatigue was prevalent in quiescent IBD patients with moderate-to-severe disease [43]. Efficacy of current interventions for fatigue (including cognitive behavioral therapy and pharmacological interventions) was inconclusive, highlighting the need for further research in this area [44].

PROs are of increasing importance given the shift to a more patient-centered approach towards IBD management. In fact, Selecting Therapeutic Targets in Inflammatory Bowel Disease (STRIDE)-II recommends the inclusion of PROs as an important treatment target in addition to clinical and endoscopic outcomes [45]. Our study serves to highlight current unmet needs in the management of UC across a varying disease severity spectrum. Over the past decade, many treatment options have emerged for the management of moderate-to-severe UC. However, our study finds that even in mild active disease, QOL and work productivity are substantially affected by UC. This highlights the need for improvement in the management of patients even at the mild end of the disease severity spectrum.

In our study, each PRO was assessed by multiple questions [16], which provides a deeper insight into the relationship between mild stages of UC and patient-reported QOL. Although the nine PRO assessment methods chosen in this study are all well established and/or validated [31, 46], the use of all these PRO tools may not be feasible in clinical practice. The IBD Disability Index was developed to measure disability in patients with IBD in daily practice [47]. The IBD Disk, a self-administered adaption of the IBD Disability Index, is an example of a valid and reliable tool for quantifying and monitoring IBD-related disability [4, 48]. The IBD Disk can be used to track changes in disease burden, and therefore it may be able to help in identifying patient-reported disability-related issues in the clinical setting [4].

The PRO scores of patients who had undergone proctocolectomy should be interpreted carefully. First, the sample size for patients who had undergone proctocolectomy was small (*n* = 42). Second, 12 (28.6%) of the 42 patients were receiving advanced therapies. This suggests that patients who had favorable surgery outcomes may be underrepresented in this study, possibly due to their less frequent hospital visits which may not have been captured during the 6-month enrolment period of this study. Despite these limitations, in all nine measures, patients who had undergone proctocolectomy had scores closer to those obtained in patients in remission than patients who had active disease. Whereas bowel symptoms or systemic symptom scores in SIBDQ were comparable in patients who had undergone proctocolectomy and patients in remission, emotional function and social function scores were lower in the patients who had undergone proctocolectomy. The reasons for these results should be explored in future studies.

The strengths of our study are (1) consecutive enrolment and large study size (more than 2000) and (2) usage of validated scales for all PROs. Conversely, the assessment of disease activity in our study lacked objective measures (endoscopy or fecal calprotectin). Although PRO-2 is reported to have significant correlation with UC disease activity based on endoscopic and histological features [49], it is solely based on patient self-reporting. Despite having a large patient cohort, patients were enrolled from a small number of investigation sites from the central region of Japan and thus may not represent the wider patient population in Japan. Patient numbers were also unequally distributed across symptom severity.

Further subgroup analyses on the correlation between PROs and symptom severity are required. Longitudinal evaluation of the relationships between disease status change and changes in PROs would be of value. Furthermore, evaluation of the relationships between PROs and types of therapeutic drug is of importance.

In summary, this study is the first large-scale prospective study assessing the challenges affecting PROs of patients with UC in Japan. The baseline data at the initial visit of this study demonstrated that many PROs were affected by UC symptom severity and proctocolectomy. There was a consistent trend of increasing impact on PROs with increasing symptom severity. The impact on PROs was found even in mild UC, suggesting that management of UC may improve PROs at all stages of disease severity.

### Supplementary Information

Below is the link to the electronic supplementary material.

Supplementary file1 (PDF 164 KB)

## Abbreviations

BMI: Body mass index
FACIT-F: Functional Assessment of Chronic Illness Therapy – Fatigue
HADS: Hospital Anxiety and Depression Scale
HCP: Healthcare providers
IBD: Inflammatory bowel disease
IPAQ: International Physical Activity Questionnaire
IQR: Interquartile range
JPSS: Japanese version of the Perceived Stress Scale
MET: Metabolic Equivalent Task
mMOS-SS: Modified Medical Outcomes Study Social Support Survey
NRS: Numerical Rating Scale
PRO: Patient-reported outcome
PRO-2: Two-item Patient Reported Outcomes
PSQI: Pittsburgh Sleep Quality Index
QOL: Quality of life
SIBDQ: Short Inflammatory Bowel Disease Questionnaire
SMD: Standardized mean difference
STRIDE: Selecting Therapeutic Targets in Inflammatory Bowel Disease
TNF: Tumor necrosis factor
UC: Ulcerative colitis
WPAI: Work Productivity and Activity Impairment
YOURS: YOu and Ulcerative colitis: Registry and Social network
