# Correction: Association of ulcerative colitis symptom severity and proctocolectomy with multidimensional patient-reported outcomes: a cross-sectional study

**DOI:** 10.1007/s00535-024-02116-9

**Published:** 2024-05-30

**Authors:** Katsuyoshi Matsuoka, Hajime Yamazaki, Masakazu Nagahori, Taku Kobayashi, Teppei Omori, Yohei Mikami, Toshimitsu Fujii, Shinichiro Shinzaki, Masayuki Saruta, Minoru Matsuura, Takayuki Yamamoto, Satoshi Motoya, Toshifumi Hibi, Mamoru Watanabe, Jovelle Fernandez, Shunichi Fukuhara, Tadakazu Hisamatsu

**Affiliations:** 1 Division of Gastroenterology and Hepatology, Department of Internal Medicine, Toho University Sakura Medical Center, 564-1 Shimoshizu, Sakura, Chiba 285-8741 Japan; 2 Section of Clinical Epidemiology, Department of Community Medicine, Graduate School of Medicine, Kyoto University, Kyoto, Japan; 3 Department of Gastroenterology and Hepatology, Tokyo Medical and Dental University, Tokyo, Japan; 4 Center for Advanced IBD Research and Treatment, Kitasato University Kitasato Institute Hospital, Tokyo, Japan; 5 Institute of Gastroenterology, Tokyo Women’s Medical University, Tokyo, Japan; 6 Division of Gastroenterology and Hepatology, Department of Internal Medicine, Keio University School of Medicine, Tokyo, Japan; 7 Department of Gastroenterology and Hepatology, Osaka University Graduate School of Medicine, Osaka, Japan; 8 Division of Gastroenterology and Hepatology, Department of Internal Medicine, The Jikei University School of Medicine, Tokyo, Japan; 9 Department of Gastroenterology and Hepatology, Kyorin University School of Medicine, Tokyo, Japan; 10 Inflammatory Bowel Disease Center and Department of Surgery, Yokkaichi Hazu Medical Center, Mie, Japan; 11 Inflammatory Bowel Disease Center, Sapporo Kosei General Hospital, Hokkaido, Japan; 12 Advanced Research Institute, Tokyo Medical and Dental University, Tokyo, Japan; 13 Japan Medical Office, Takeda Pharmaceutical Company Limited, Tokyo, Japan; 14 Department of Health Policy Management, Johns Hopkins Bloomberg School of Public Health, Maryland, USA; 15 Present Address: Division of Gastroenterology and Hepatology, Department of Internal Medicine, Hyogo Medical University, Hyogo, Japan

**Correction: Journal of Gastroenterology (2023) 58:751–765** 10.1007/s00535-023-02005-7

Fig. 2h of the original article contained extraneous yellow data bars; Fig. 2h should appear as shown in this correction.

**Fig. 2:**
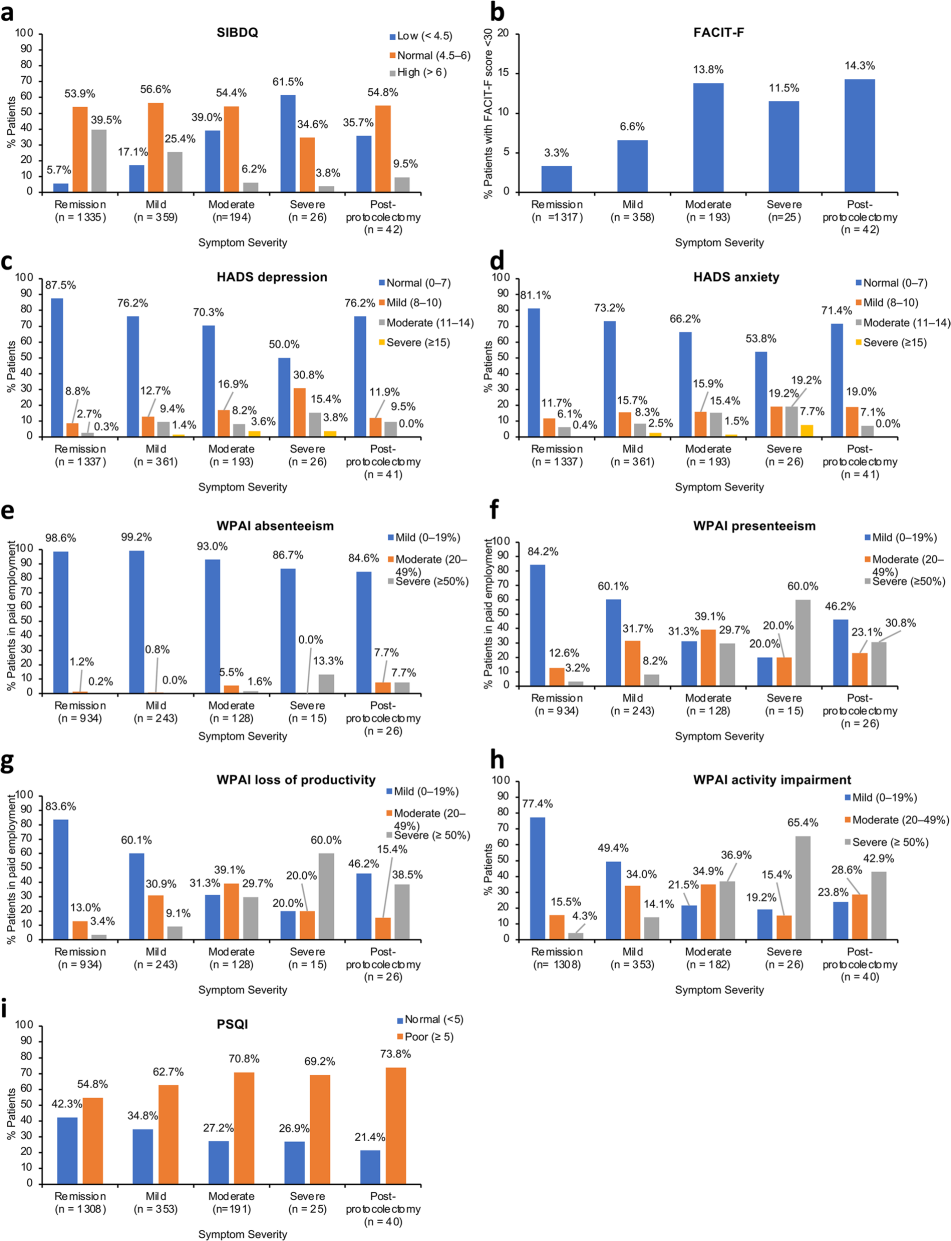
Proportion of patients reporting **a** QOL as low, normal or high on SIBDQ; **b** severe fatigue on FACIT-F; **c** depression or **d** anxiety on the HADS scale; **e** absenteeism, **f** presenteeism, **g** work productivity, and **h** activity impairment on WPAI; and **i** poor sleep quality on PSQI. *FACIT-F* Functional Assessment of Chronic Illness Therapy – Fatigue, *HADS* Hospital Anxiety and Depression Scale, *PSQI* Pittsburgh Sleep Quality Index, *QOL* quality of life, *SIBDQ* Short Inflammatory Bowel Disease Questionnaire, *WPAI* Work Productivity and Activity Impairment

In Fig. 3 of the original article, the labelling of ‘y’ axis was reversed (i.e., better/worse outcome); Fig. 3 should appear as shown in this correction.

**Fig. 3:**
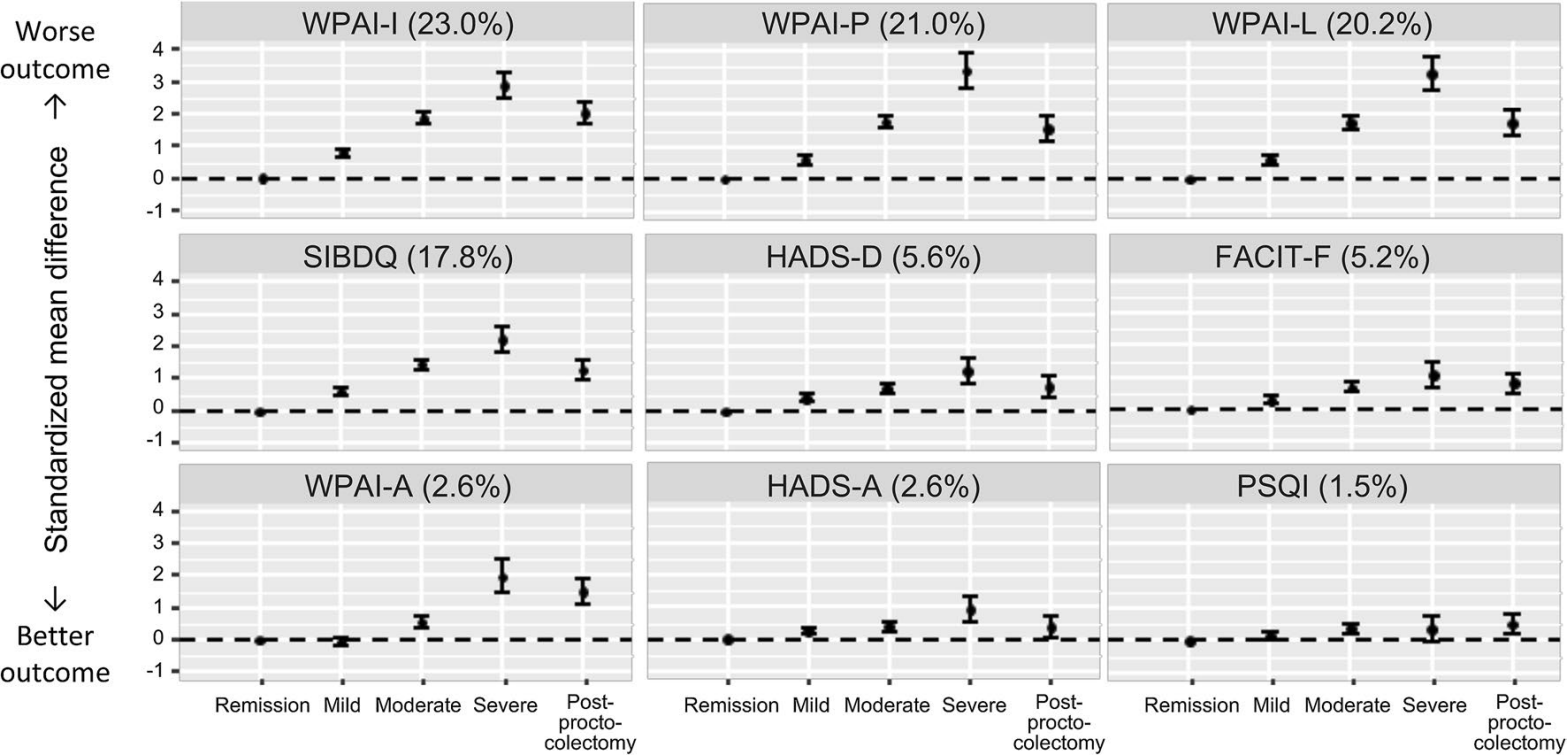
Associations of symptom severity and proctocolectomy with patient-reported outcomes. Data indicate SMD and their 95% confidence intervals. The fraction of variance of symptom severity explained by each outcome is shown in parentheses. The magnitude of the effect size was interpreted as: small, SMD = 0.2; medium, SMD = 0.5; and large, SMD = 0.8 [39]. Total number of patients included in the analysis were: SIBDQ (*N* = 1956), FACIT-F (*N* = 1935), HADS-D (*N* = 1958), HADS-A (*N* = 1958), WPAI-A (*N* = 1346), WPAI-P (*N* = 1346), WPAI-L (*N* = 1346), WPAI-I (*N* = 1909), and PSQI (*N* = 1917) (see Table 3 footnotes for breakdown by patient group). *FACIT-F* Functional Assessment of Chronic Illness Therapy – Fatigue, *HADS* Hospital Anxiety and Depression Scale (A, anxiety; D, depression), *PSQI* Pittsburgh Sleep Quality Index, *SIBDQ* Short Inflammatory Bowel Disease Questionnaire, *SMD* standardized mean difference, *WPAI* Work Productivity and Activity Impairment (A, absenteeism; I, impairment of activity; L, loss of productivity; P, presenteeism)

