# Supplementary material for: Association of ulcerative colitis symptom severity and proctocolectomy with multidimensional patient-reported outcomes: a cross-sectional study
Source: J Gastroenterol. 2023 Jun 23;58(8):751–65. doi: 10.1007/s00535-023-02005-7 (PMC10366259; doi:10.1007/s00535-023-02005-7)

Supplementary Information

Association of ulcerative colitis symptom severity and proctocolectomy with multidimensional patient-reported outcomes: a cross-sectional study

Katsuyoshi Matsuoka[1] · Hajime Yamazaki[2] · Masakazu Nagahori[3] · Taku Kobayashi[4] · Teppei Omori[5] · Yohei Mikami[6] · Toshimitsu Fujii[3] · Shinichiro Shinzaki[7] · Masayuki Saruta[8] · Minoru Matsuura[9] · Takayuki Yamamoto[10] · Satoshi Motoya[11] · Toshifumi Hibi[4] · Mamoru Watanabe[12] · Jovelle Fernandez[13] · Shunichi Fukuhara[2,14] · Tadakazu Hisamatsu[9]

1 Division of Gastroenterology and Hepatology, Department of Internal Medicine, Toho University Sakura Medical Center, Chiba, Japan
2 Section of Clinical Epidemiology, Department of Community Medicine, Graduate School of Medicine, Kyoto University, Kyoto, Japan
3 Department of Gastroenterology and Hepatology, Tokyo Medical and Dental University, Tokyo, Japan
4 Center for Advanced IBD Research and Treatment, Kitasato University Kitasato Institute Hospital, Tokyo, Japan
5 Institute of Gastroenterology, Tokyo Women's Medical University, Tokyo, Japan
6 Division of Gastroenterology and Hepatology, Department of Internal Medicine, Keio University School of Medicine, Tokyo, Japan
7 Department of Gastroenterology and Hepatology, Osaka University Graduate School of Medicine, Osaka, Japan
8 Division of Gastroenterology and Hepatology, Department of Internal Medicine, The Jikei University School of Medicine, Tokyo, Japan
9 Department of Gastroenterology and Hepatology, Kyorin University School of Medicine, Tokyo, Japan
10 Inflammatory Bowel Disease Center & Department of Surgery, Yokkaichi Hazu Medical Center, Mie, Japan
11 Inflammatory Bowel Disease Center, Sapporo Kosei General Hospital, Hokkaido, Japan
12 TMDU Advanced Research Institute, Tokyo Medical and Dental University, Tokyo, Japan
13 Japan Medical Office, Takeda Pharmaceutical Company Limited, Tokyo, Japan
14 Department of Health Policy Management, Johns Hopkins Bloomberg School of Public Health, Maryland, USA

Correspondence:

Katsuyoshi Matsuoka, MD. PhD.
Address: Toho University Sakura Medical Centre, 564-1 Shimoshizu, Sakura, Chiba 285-8741, Japan
Phone: +81-43-462-8811
Fax: +81-43-487-4246
Email: matsuoka@fk2.so-net.ne.jp
ORCID: https://orcid.org/0000-0002-2950-7660

Supplemental Table 1 Social and lifestyle factors by disease severity

Factors	Remission (n = 1346)	Mild (n = 362)	Moderate (n = 195)	Severe (n = 26)
Socioeconomic status				
Employment, n (%)				
Student	91 (6.8)	27 (7.5)	16 (8.2)	1 (3.8)
Unemployed	275 (20.4)	81 (22.4)	39 (20.0)	10 (38.5)
Employed	971 (72.1)	252 (69.6)	140 (71.8)	15 (57.7)
Missing	9 (0.7)	2 (0.6)	0	0
Annual income (JPY), n (%)				
< 3 million	160 (11.9)	63 (17.4)	26 (13.3)	6 (23.1)
≥ 3 and < 5 million	327 (24.3)	108 (29.8)	48 (24.6)	11 (42.3)
≥ 5 and < 7 million	278 (20.7)	66 (18.2)	40 (20.5)	5 (19.2)
≥ 7 and < 10 million	260 (19.3)	63 (17.4)	37 (19.0)	2 (7.7)
≥ 10 and < 12 million	131 (9.7)	22 (6.1)	13 (6.7)	1 (3.8)
≥ 12 million	145 (10.8)	37 (10.2)	24 (12.3)	1 (3.8)
Missing	45 (3.3)	3 (0.8)	7 (3.6)	0
Education, n (%)				
Junior high school	22 (1.6)	9 (2.5)	3 (1.5)	4 (15.4)
High school	217 (16.1)	62 (17.1)	37 (19.0)	8 (30.8)
Vocational school	171 (12.7)	51 (14.1)	32 (16.4)	2 (7.7)
Community college	117 (8.7)	29 (8.0)	9 (4.6)	1 (3.8)
University	535 (39.7)	135 (37.3)	75 (38.5)	9 (34.6)
Graduate school	82 (6.1)	24 (6.6)	7 (3.6)	0
Missing	202 (15.0)	52 (14.4)	32 (16.4)	2 (7.7)
Social factors				
Social support (mMOS-SS), median (IQR)[a]	75.00 (31.25)	71.88 (37.50)	71.88 (34.38)	68.75 (46.88)
Stress (JPSS), median (IQR)[b]	23.0 (9.0)	25.0 (10.0)	26.0 (11.0)	29.0 (5.0)
Living alone, n (%)	211 (15.7)	58 (16.0)	29 (14.9)	8 (30.8)

[a]Remission (n = 1342), Mild (n = 361), Moderate (n = 194), Severe (n = 26); [b]Remission (n = 1328), Mild (n = 359), Moderate (n = 193), Severe (n = 26).
IQR, interquartile range; JPSS, Japanese version of the Perceived Stress Scale; mMOS-SS, modified Medical Outcomes Study Social Support Survey.

Supplementary Fig. 1 Disposition of patients

PRO-2, Two-item Patient Reported Outcomes; YOURS, YOu and Ulcerative colitis: Registry and Social network.

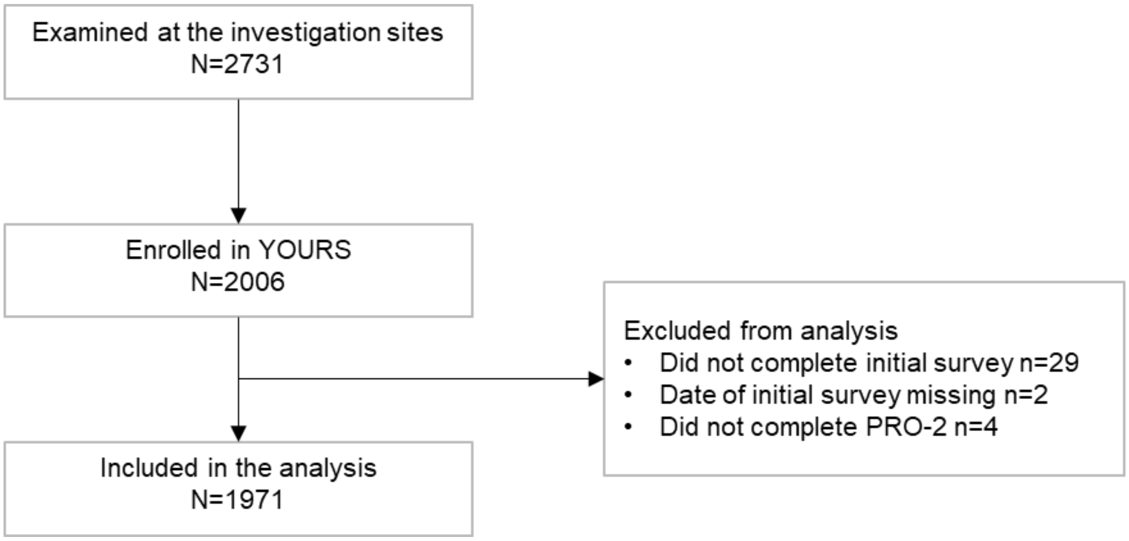

Supplementary Fig. 2 Hierarchical clustering analysis of patient-reported outcomes

For SIBDQ and FACIT-F, sign of the standardized scores were flipped so that negative and positive values would correspond to better and worse symptoms, respectively. Patients in remission and those with active disease (mild, moderate, or severe) who were assessable for all nine PROs were included in the analysis (N = 1263). FACIT-F, Functional Assessment of Chronic Illness Therapy – Fatigue; HADS, Hospital Anxiety and Depression Scale (A, anxiety; D, depression); PRO, patient-reported outcome; PSQI, Pittsburgh Sleep Quality Index; SIBDQ, Short Inflammatory Bowel Disease Questionnaire; WPAI, Work Productivity and Activity Impairment (A, absenteeism; I, impairment of activity; L, loss of productivity; P, presenteeism).

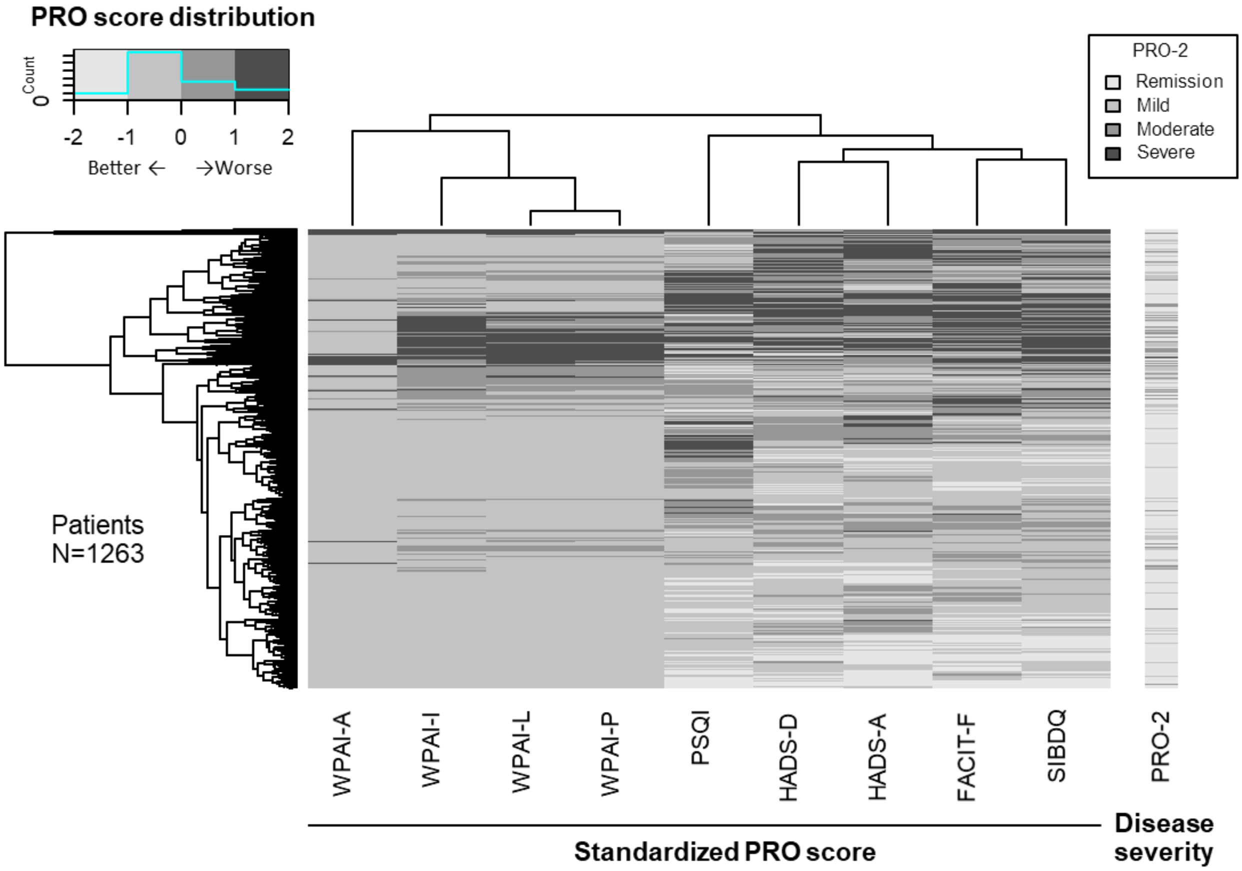

Supplement: Supplementary file 1 — Supplementary file1 (PDF 164 KB) [file 535_2023_2005_MOESM1_ESM.pdf]
